# Active transcutaneous bone-anchored hearing implant: how I do it

**DOI:** 10.1007/s00405-021-06946-8

**Published:** 2021-06-21

**Authors:** S. Arndt, A. K. Rauch, I. Speck

**Affiliations:** Department of Oto-Rhino-Laryngology, Medical Center–University of Freiburg, Killianstraße 5, 79105 Freiburg, Germany

**Keywords:** Bone conduction hearing, Mixed hearing loss, Single-sided deafness, BAHI, OSIA, Auditory prosthesis

## Abstract

**Background:**

The Cochlear™ Osia® System leaves a retroauricular bump that can cause discomfort and poor aesthetic outcome.

**Method:**

To reduce the retroauricular bump, we introduced an implant well in the bone behind the ear for the transducer. We used cutting and diamond drills to create the implant well with an average depth of 4–5 mm. The surgical time including the implant well (40 min) was within the range of reported average surgical time (52 min).

**Conclusion:**

Introduction of an implant well resulted in a better aesthetic outcome and improved patients’ comfort. The reduced distance between BI300 and ear canal might improve audiological outcome.

**Supplementary Information:**

The online version contains supplementary material available at 10.1007/s00405-021-06946-8.

## Background

The Cochlear™ Osia® System (Osia; Cochlear, Sydney, Australia) is a new generation of an active transcutaneous bone-anchored hearing implant with a newly developed piezoelectric transducer that is fixed to a titanium implant (BI300). The implant is indicated in patients with unilateral and bilateral conductive or mixed hearing loss and single-sided deafness. The integrated digital piezoelectric stimulation allows for bone conduction hearing loss of up to 55 dB [1]. Compared to the Baha®5 Power, the Osia® System shows a significantly higher functional gain in higher frequencies (5–7 kHz) [2].

**Fig. 1:**
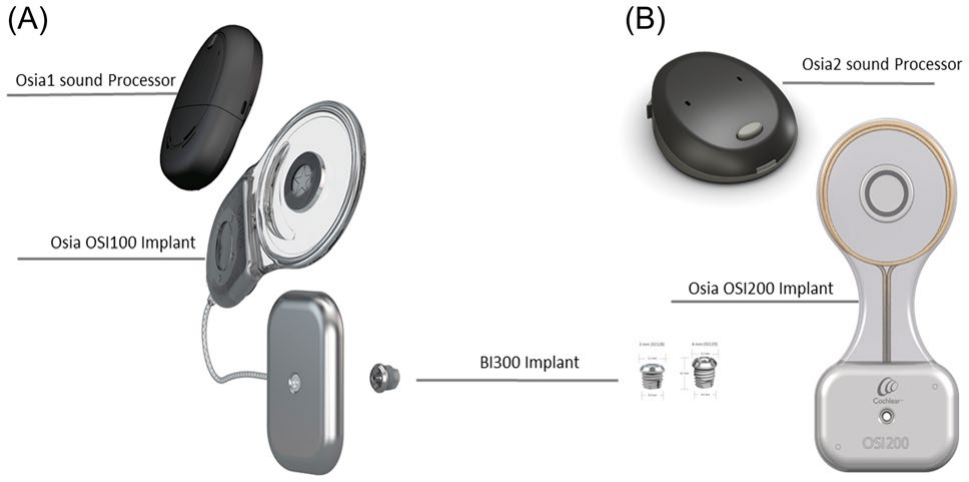
Cochlear™ Osia® System implants OSI100 and OSI200 with audio processor (Osia; Cochlear, Sydney, Australia)

Recently, the second generation OSI200 was released. Unlike the first-generation transducer OSI100, the OSI200 has a stable connection between transducer and coil allowing for less flexibility regarding transducer and coil position to reduce feedback noises (Fig. 1).

**Fig. 2:**
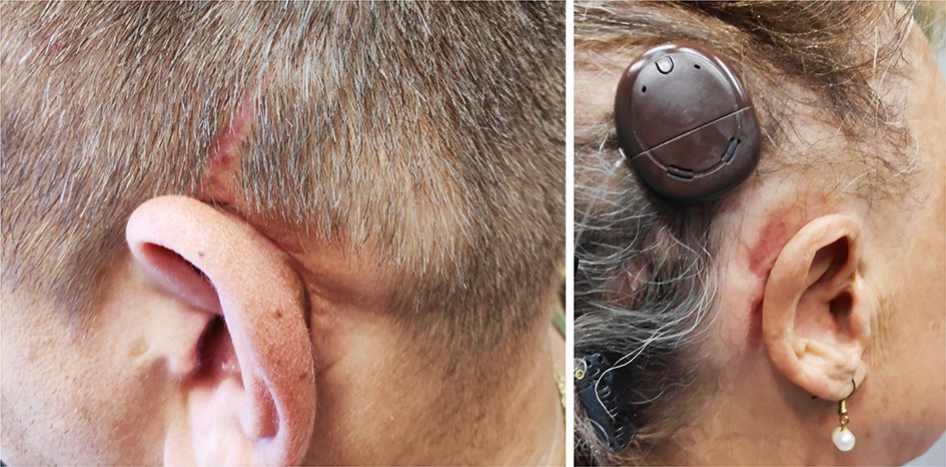
Patients with retroauricular bump of the Osia® implant

We have implanted 29 OSI100 and 7 OSI200 to date. Following our first implantations, we were confronted with the following complaints of the patients: First, patients were unsatisfied with the aesthetic outcome due to the consequent bump of the Osia® implant (Fig. 2). Second, as nearly all implanted patients wear glasses—at least sunglasses—patients reported pain and pressure marks when the temples were near or in contact with the transducer. This complication was also reported by Lau et al. [3]

To reduce these complaints, we needed a surgical method to implant the OSI that led to a smaller or absent bump behind the patient’s ear.

Following company recommendation for implantation of the OSI no bony recess for the transducer is necessary. Merely, the BI300 should be implanted in the skull. The transducer, with a thickness of 4.9 mm, should not be in contact with the skull bone and, therefore, the position of the transducer is clearly visible and palpable behind the patient’s ear (Fig. 2). As a consequence, we changed our surgical approach to achieve a reduction of the bulk behind the ear by preparing an implant well for the transducer of the OSI.

### Preoperative workup

**Fig. 3:**
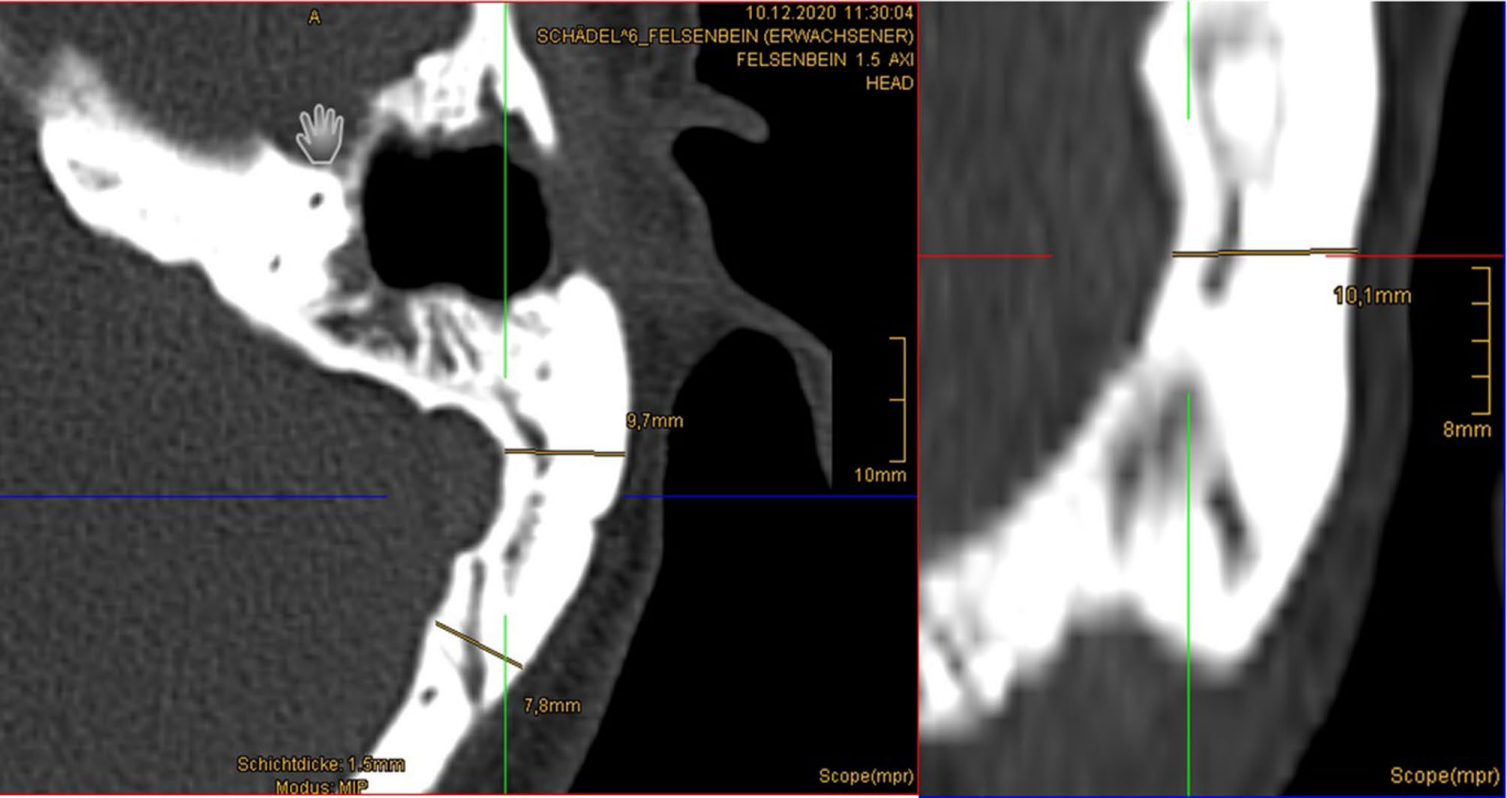
Digital volume tomography of the temporal bone is necessary with measured bone thickness behind the ear at implantation site of the titanium fixture

To determine the possible depth of the bony recess, a preoperative computer tomography (CT) or digital volume tomography (DVT) of the temporal bone is necessary. As nearly all of the patients had previous ear surgeries, temporal bone CT scans were mostly available. The minimal required bone thickness behind the ear canal was 3 mm for the implantation if the titanium fixture (Fig. 3). To level the top of the OSI to the bone surface, a recess of at least 9 mm would be necessary (thickness of implant of 4.9 mm plus app. 1 mm distance between implant and bone surface and 3 mm BI300 implant). Therefore, a complete lowering of the implant is mostly not possible, but at least partial lowering can be achieved.

### Surgical technique

**Fig. 4:**
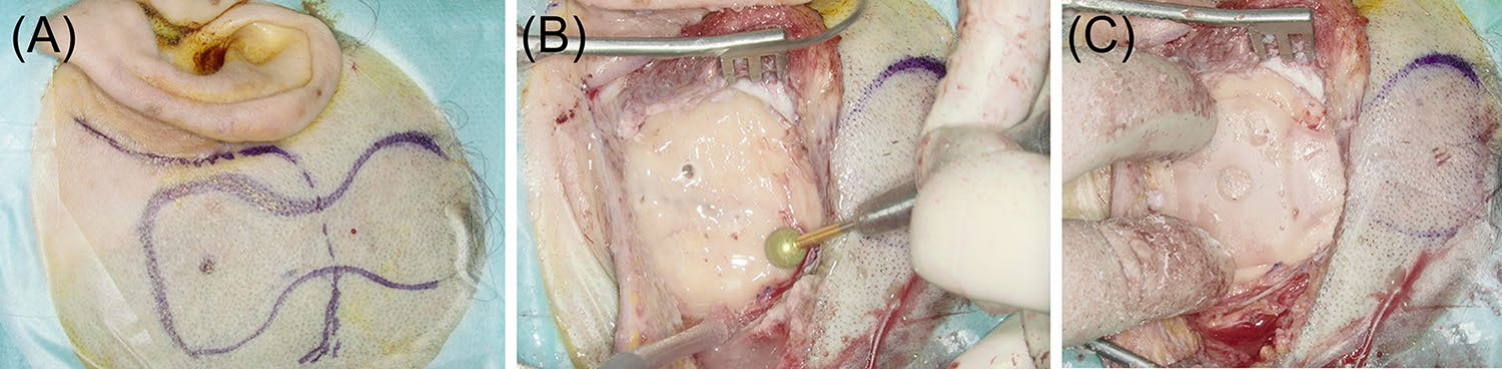
Surgical steps preparation of the implant well: **A** planned incision and position of the OSI200. **B** Preparation of the implant well. **C** Checking depth and shape of the implant well with the implant template

Surgeries were performed under general anesthesia. Intraoperative preparation and incision were performed as suggested by the guidelines of the manufacturer. We marked the planned incision and position of the OSI100 or OSI200 on the skin (Fig. 4A). The skin thickness over the implant coil was measured and only thinned if the skin thickness was above 9 mm. The incision of the skin and periost was performed in different positions to minimize scaring and possible infections.

Following clearance of the periosteum, we deviated from the guidelines of the manufacturer. We marked the location of the BI300 Implant and transducer on the skull bone. The implant well in the bone behind the ear was prepared using drills (Fig. 4B). The depth of the recess was based on the preoperative workup using CT or DVT. In most surgeries, we prepared an implant well of 4–5 mm to lower the implant; a levelling to the bone surface with a bony recess of 9 mm was only rarely possible. The implant template was used to check depth and shape of the implant well (Fig. 4C). In case of the OSI200, the implant well was extended in all directions to create a soft bony ramp for the fixed connection between transducer and coil. Before implanting the BI300, the edges of the recess were smoothened.

**Fig. 5:**
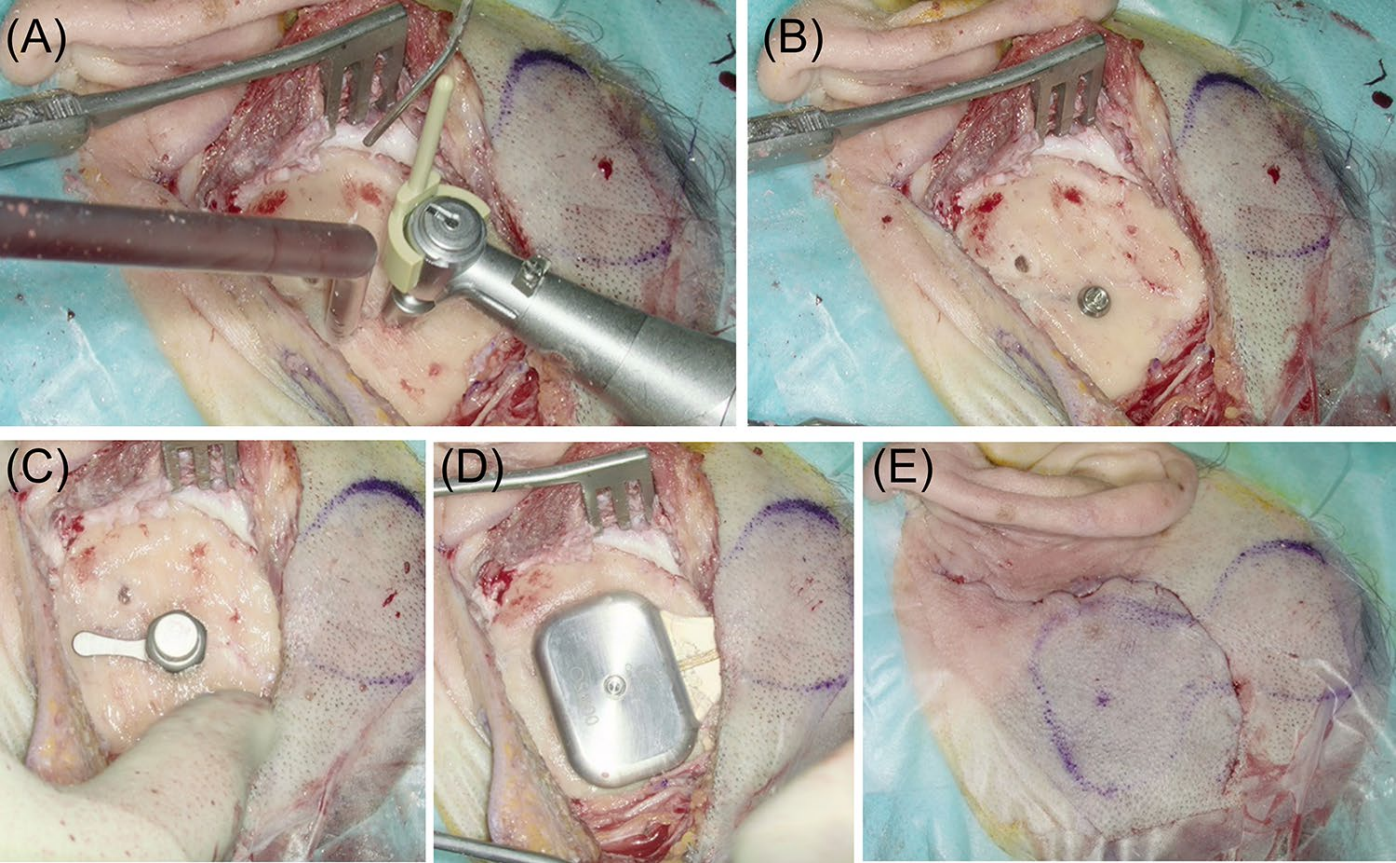
Surgical steps of the OI200 implantation: **A** drilling with the countersink drill. **B** Placing of the BI300. **C** Checking clearance with clearance indicator. **D** Placing of the OSI200. **E** Operation situs after closing incision

After preparation of the recess, the guidelines of the manufacturer were followed again. We used the 3 mm guide drill to create a hole, which was than widened with the countersink drill (Fig. 5A) and the BI300 implant was placed in the bone with 40Ncm (Fig. 5B). Before inserting the OSI, the clearance indicator was used to check for interfering bone. The transducer and consequently the implant well are rectangular. Therefore, the clearance indicator, that covers a circular shape, will come in contact with the edges of the implant well (Fig. 5C). This contact does not indicate insufficient clearance as the transducer will levitate on the BI300 in the prepared implant well. Alternatively, the smaller clearance indicator from Baha attract surgery can be used.

Then, we placed the center of the transducer on top of the BI300 and tighten the fixation screw with 25Ncm (Fig. 5D). Due to the preoperative planning of the possible implantation depth, we have never experienced any problems with the fixation of the BI300 implant so far. Following implantation, the skin flap is closed over the implant using multi-layer sutures (Fig. 5E).

### Surgical outcome and discussion

Our surgical time from incision to closure varied between 30 and 60 min. Goldstein et al. [4] reported an average surgical time of 52 min without implant well preparation.

**Fig. 6:**
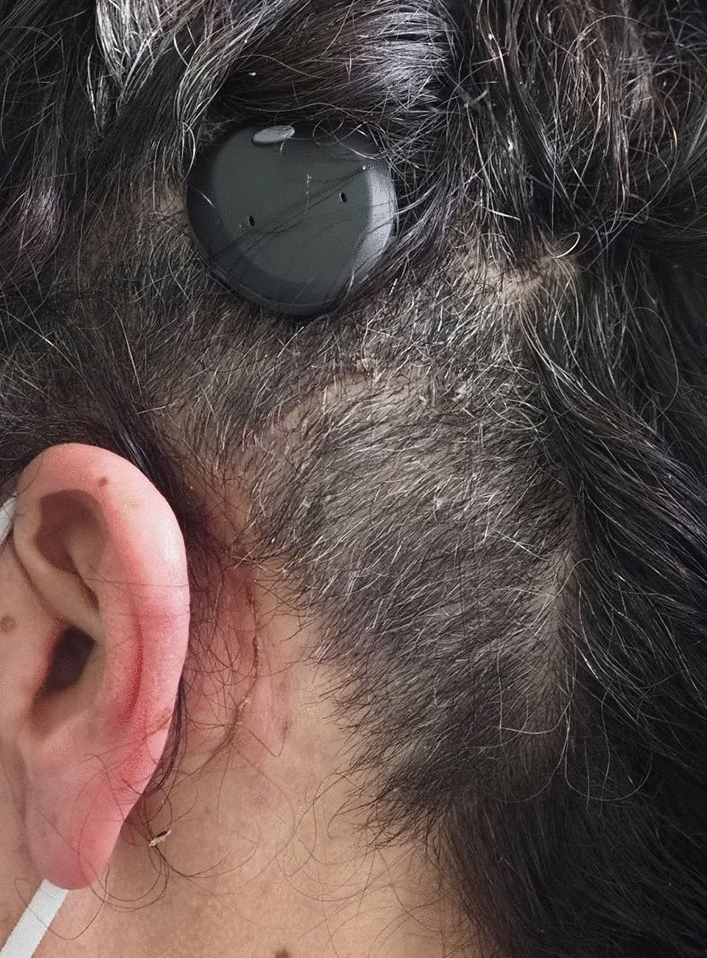
Patient with minimal retroauricular bump of the Osia® implant after surgery including the implant well

After introduction of the modified surgical method including an implant well, the transducer-bump was significantly reduced (Fig. 6). Patients expressed a greater satisfaction with the aesthetic outcome and had no problems and experienced no pain wearing their glasses. Discomfort and pain caused by the implant were reported less.

In addition to the advantages in aesthetic and patient comfort, our surgical technique also possesses an audiological advantage. The lowering of the transducer in the bone can lead to a reduced distance between BI300 and the auditory canal in most of the patients. Eeg-Olofsson et al. [5] reported higher cochlear promontory acceleration with closer stimulation to the ear canal opening. This was confirmed by Reinfeldt et al. [6] showing an improvement in hearing threshold and ear canal sound pressure. Therefore, improved hearing thresholds can be expected with the installation of an implant well for the OSI200.

We recommend introduction of an implant well for the OSI implant, which resulted in both enhanced aesthetic outcome and patient comfort. In addition, the implant well led to a reduced distance of the implant to the auditory canal, enabling improved audiological outcome.

### Surgical key points

1. An implant well in the bone behind the ear for the transducer of the OSI200 can reduce the retroauricular bump.
2. To plan the depths of the recess, a presurgical computer tomography (CT) or digital volume tomography (DVT) is necessary to determine bone thickness.
3. To level the transducer to the skull-surface, a bone thickness of 9 mm is necessary: BI300 3 mm plus transducer height of 4.9 mm plus 1 mm distance between transducer and bone surface.
4. In average, a recess of a depth of 4–5 mm was installed.
5. The clearance indicator will come in contact to the bone because of the rectangular shape of transducer and consequently, the bone edges of the implant well. This contact does not indicate insufficient clearance. Alternatively, the smaller clearance indicator from Baha attract surgery can be used for this purpose only.
6. The surgical time is with a mean of 40 min in the range of reported average surgical time of 52 min.
7. The installation of a recess results in a better aesthetic outcome and improved patients’ comfort.
8. The reduced distance between BI300 and ear canal might lead to improved audiological outcome.

## Supplementary Information

Below is the link to the electronic supplementary material.

Supplementary file1 (MP4 365224 KB)
